# Adolescent prodromal phenotypes of psychiatric disorders: insights from a novel DISC1-perturbed monkey model

**DOI:** 10.64898/2026.10.11.758339

**Published:** 2026-10-18

**Authors:** Ziguo Lan, Hirotaka Onoe, Tomoyuki Tsukiyama, Chizuru Iwatani, Chih-Yang Chen, Keisuke Okita, Koko Ishizuka, Ryo Endo, Risako Nakai, Keiko Imamura, Keigo Hikishima, Hideaki Tsuchiya, Masataka Nakaya, Yang Fang, Atsushi Kobayashi, Yuta Shinto, Reona Yamaguchi, Mami Matsunaga, Kengo Oe, Koichi Omori, Yuji Ozeki, Toshifumi Tomoda, Kenichiro Miura, Shin-ichi Urayama, Naoya Oishi, Tomohisa Okada, Motomasa Tanaka, Haruhisa Inoue, Jun Takahashi, Akira Sawa, Tadashi Isa

## Abstract

Neurodevelopmental deficits frequently lead to mild but significant adolescent social and cognitive changes, representing prodromal manifestations of adult-onset psychiatric disorders in a cross-disease manner. Although experimental medicine utilizes animal models to address the mechanisms, rodent brains lack critical structures for human higher brain functions such as the dorsolateral prefrontal cortex. To fill this translational gap, we generated monkeys with an impaired *DISC1* gene locus, targeting the multifunctional protein essential for neurodevelopment and involved in transdiagnostic mental health risks. Here we analyze the F0 animals by minimizing mosaicism. At ages equivalent to human adolescence, these monkeys exhibited blunted responsiveness to social stimuli and increased internal drives, mirroring altered social behaviors in the prodromal stage in humans. Resting-state functional MRI data indicated disrupted global functional connectivity and reorganization of network topology, marked by a shift in the dominance of brain hubs from prefrontal regions to sensory areas. This primate model provides a unique platform for studying psychiatric disorders, such as schizophrenia, across the developmental trajectory.

